# Supplementary material for: Genotoxic stress-triggered β-catenin/JDP2/PRMT5 complex facilitates reestablishing glutathione homeostasis
Source: Nat Commun. 2019 Aug 21;10:3761. doi: 10.1038/s41467-019-11696-7 (PMC6704105; doi:10.1038/s41467-019-11696-7)
Supplement: Supplementary file 1 — Supplementary Information [file 41467_2019_11696_MOESM1_ESM.pdf]

Supplementary Information

Genotoxic stress-triggered β-catenin/JDP2/PRMT5 complex facilitates reestablishing glutathione homeostasis

Cao et al.

Supplementary Figure 1

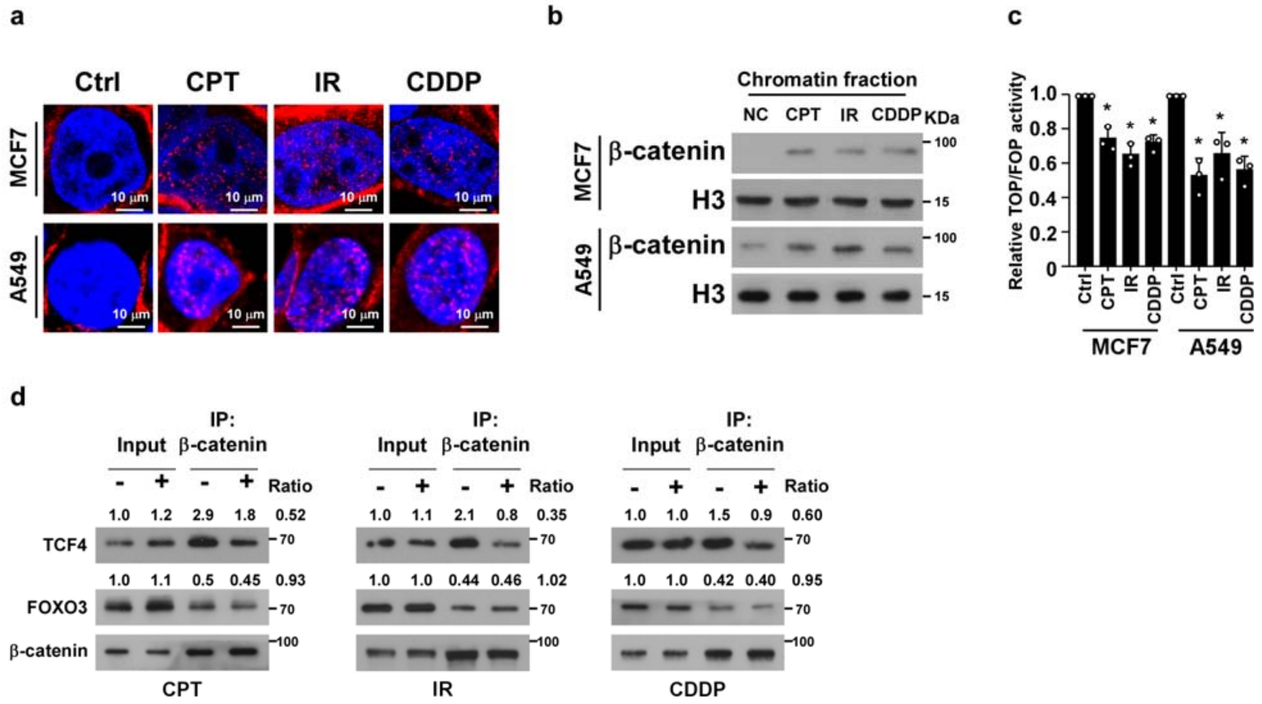

Supplementary Figure 1. β-catenin is enriched in chromatin in response to genotoxic stress. (a) Representative images of the subcellular localization of β-catenin in MCF-7 and A549 cells treated with CPT (10 μM, 1 h), IR (10 Gy), and CDDP (10 μM, 1 h), as analyzed by immunofluorescence staining. Scale bar, 10 μm. **(b)** IB analysis of β-catenin expression in the chromatin fraction extracted from the indicated cells treated with CPT (10 μM, 1 h), IR (10 Gy), and CDDP (10 μM, 1 h). Histone 3 served as the loading control. **(c)** Relative TOPflash or FOPflash luciferase reporter activity was analyzed in the indicated cells treated with CPT (10 μM, 1 h), IR (10 Gy), and CDDP (10 μM, 1 h). Each error bar represents the mean ± SD of three independent experiments. * $P < 0.05$. Student' s 2-tailed t test. **(d)** IP assays using anti-β-catenin antibody were performed in the indicated cells treated with CPT (10 μM, 1 h), IR (10 Gy), and CDDP (10 μM, 1 h), and IB analysis of the expression of β-catenin, TCF4, and FOXO3. Numbers below the panels are the quantification of the signals determined by densitometry, in which the first line was set as 1.0. The ratio was defined as + (IP: Input)/**-** (IP: Input). Source data of Supplementary Fig.1c are provided as a Source Data file.

Supplementary Figure 2

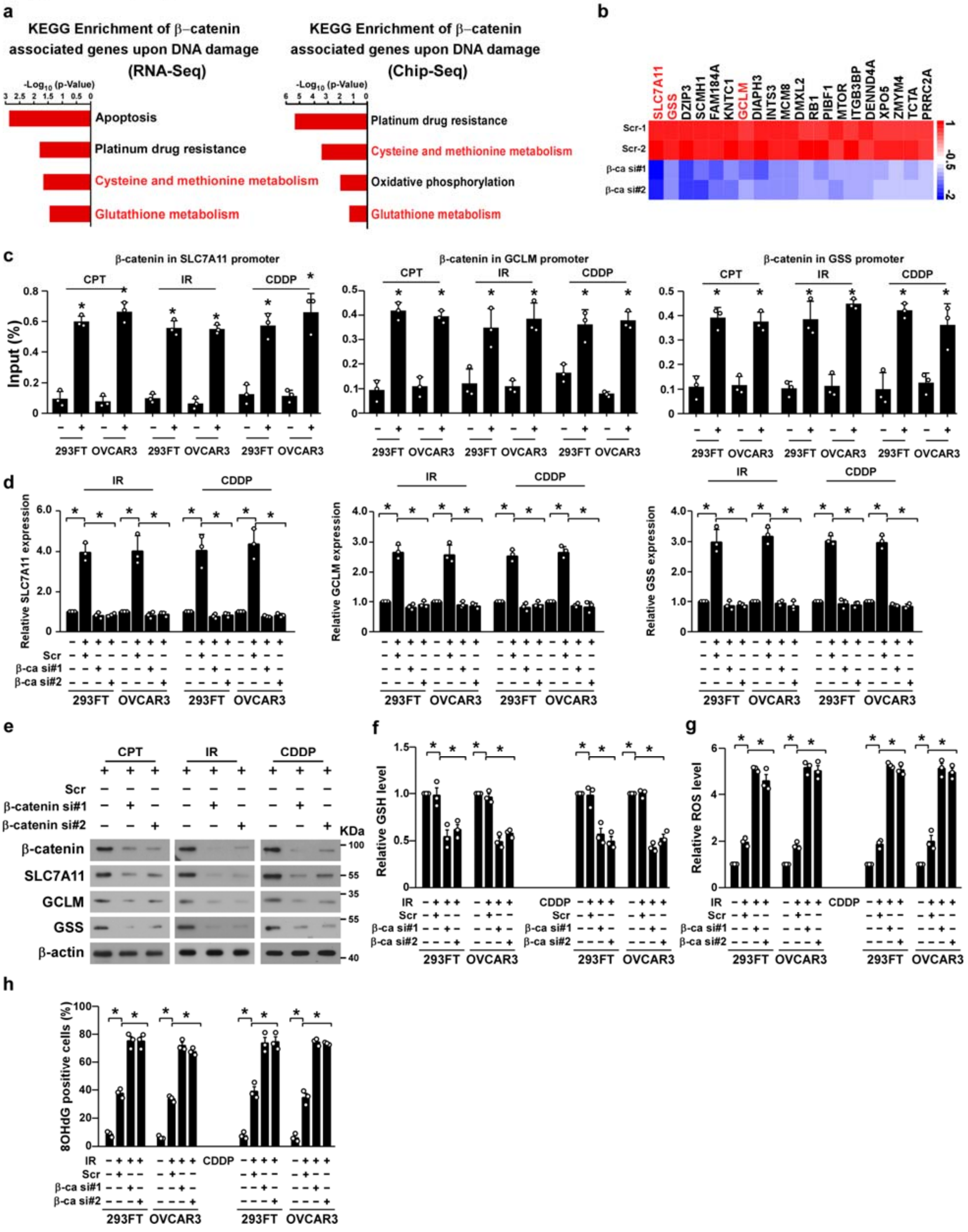

Supplementary Figure 2. Genotoxic stress-activated β-catenin signaling induced GSH metabolism via upregulation of SLC7A11, GCLM, and GSS. (a) KEGG pathway

enrichment analysis of β-catenin-regulated transcripts identified using RNA-seq (PRJNA543096, left) or ChIP-seq (PRJNA543097, right) profiling in control and β-catenin-silenced 293FT cells treated with CPT (10 μM, 1 h). The x-axis shows the enrichment scores as calculated using the –log10 (p value). **(b)** A heatmap represented by pseudocolors was generated using the RNA-Seq values in control and β-catenin silenced 293T cells treated with CPT (10 μM, 1 h), with red and green representing high and low expression levels in indicated cells. Colors were shown on a Log scale. **(c)** ChIP assay analysis of the enrichment of β-catenin on the promoters of *SLC7A11*, *GCLM*, and *GSS* in the indicated cells treated with CPT (10 μM, 1 h), or IR (10 Gy), or CDDP (10 μM, 1 h). **(d)** Relative expression of *SLC7A11*, *GCLM*, and *GSS* in IR (10 Gy)-, or CDDP (10 μM, 1 h)-treated cells, as quantified by qRT-PCR analysis. **(e)** IB analysis of the expression of SLC7A11, GCLM, and GSS proteins in CPT (10 μM, 4h)-, IR (10 Gy)-, or CDDP (10 μM, 4h)-treated cells transfected with scramble or β-catenin-siRNA(s). β-actin served as the loading control. **(f-g)** Relative levels of GSH (f) and ROS (g) were examined in scramble- or β-catenin siRNA(s)-transfected cells upon indicated treatments (3 h). **(h)** The percentage of 8OHdG-positive cells in 293FT, OVCAR3 cells treated as indicated and analyzed using 8OHdG staining. Each error bar in c, d, f and h represents the mean ± SD of three independent experiments. * $P < 0.05$. Student' s 2-tailed t test. Source data of Supplementary Fig.2c-d and 2f-h are provided as a Source Data file.

Supplementary Figure 3

a

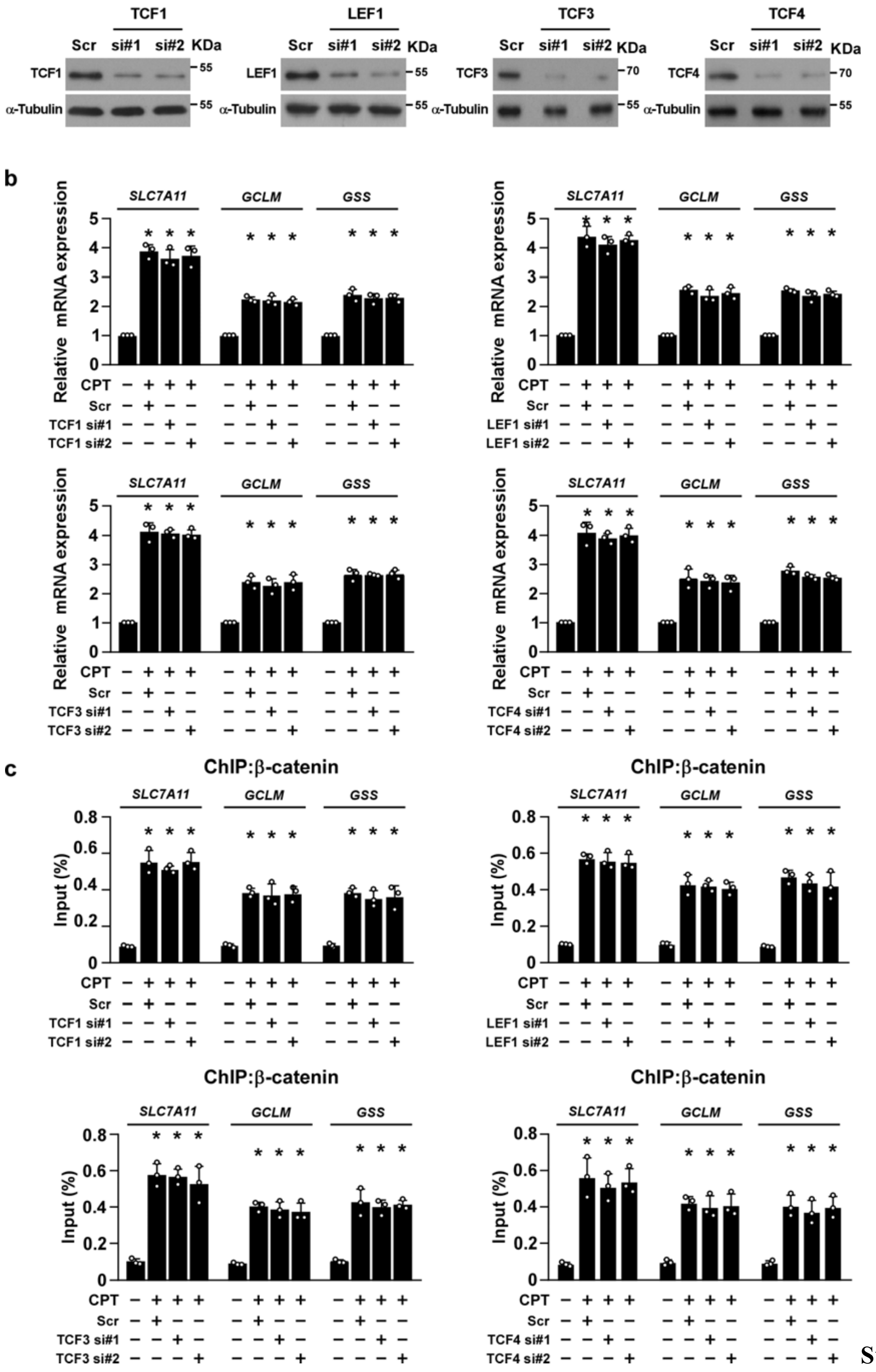

tary Figure 3. Genotoxic stress activated-β-catenin signaling is through a TCF-independent mechanism. **(a)** IB analysis of the expression of TCF1 (TCF7), LEF1, TCF3 (TCF7L1), and TCF4 (TCF7L2) in the scramble- or indicated siRNA(s)-transfected cells. α-tubulin served as the loading control. **(b)** Relative mRNA expression of *SLC7A11*, *GCLM*, and *GSS* in CPT (10 μM, 1 h)-treated cells transfected with scramble or indicated siRNA(s), as quantified by qRT-PCR analysis. *GAPDH* served as the loading control. **(c)** ChIP assay analysis of the enrichment of β-catenin on the promoter of *SLC7A11* in CPT (10 μM, 1 h)-treated cells transfected with scramble or indicated siRNA(s). Each error bar in b-c represents the mean ± SD of three independent experiments. * $P < 0.05$. Student' s 2-tailed t test. Source data of Supplementary Fig.3b-c are provided as a Source Data file.

Supplementary Figure 4

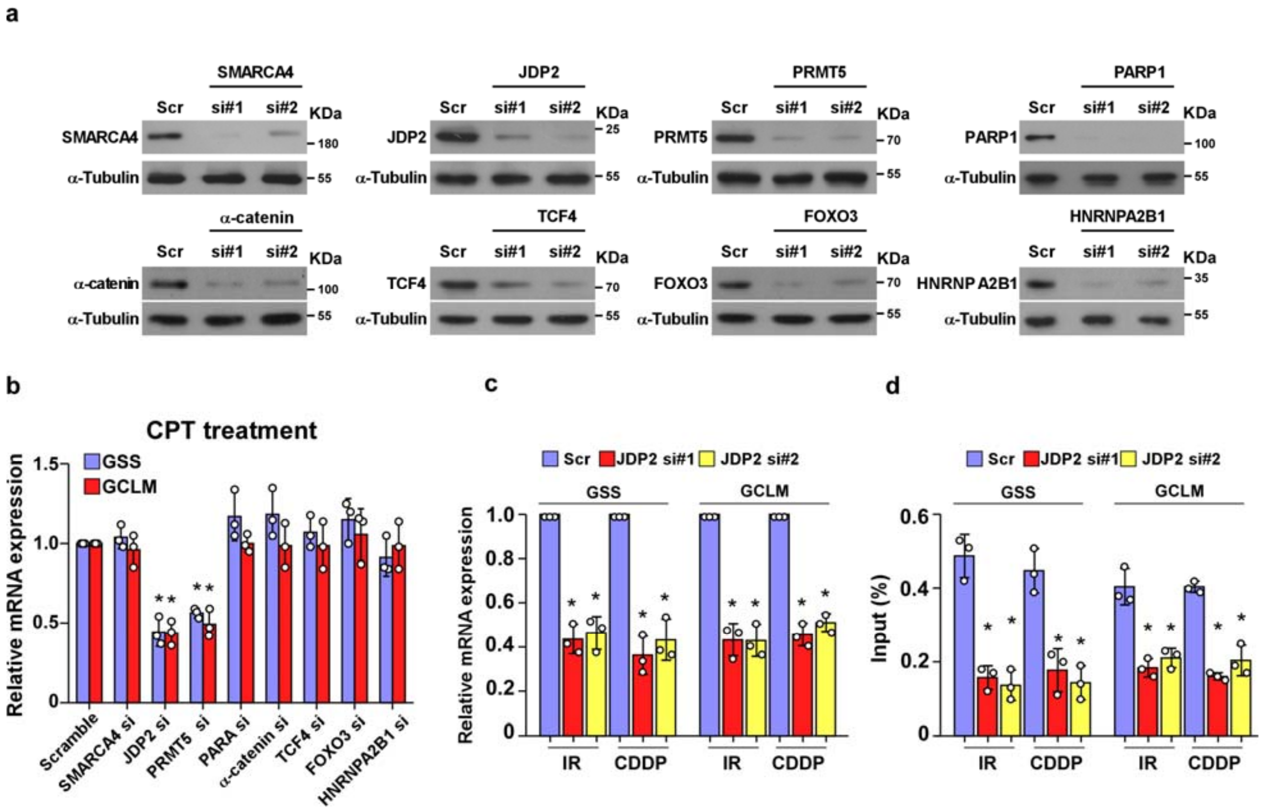

Supplementary Figure 4. JDP2 is essential for β-catenin-induced GSH metabolism upon genotoxic stress. **(a)** IB analysis of the expression of SMARCA4, JDP2, PRMT5, PARP1, α-catenin, TCF4, FOXO3 and HNRNPA2B1 in the scramble- or indicated siRNA(s)-transfected cells. α-tubulin served as the loading control. **(b)** Relative mRNA expression of *GSS* and *GCLM* in the indicated siRNA-transfected cells treated with CPT (10 μM, 1 h), as quantified by qRT-PCR analysis. *GAPDH* served as the loading control. **(c)** Relative mRNA expression of *GSS* and *GCLM* in the IR- or CDDP-treated cells transfected with scramble or JDP2 siRNA(s), as quantified by qRT-PCR analysis. *GAPDH* served as the loading control. **(d)** ChIP assays analyses of the enrichment of β-catenin on the promoters of *GSS* and *GCLM* in the IR (10 Gy)-, or CDDP (10 μM, 4h)-treated cells transfected with scramble or JDP2 siRNA(s). Each error bar in b-d represents the mean ± SD of three independent experiments. * $P < 0.05$. Student' s 2-tailed t test. Source data of Supplementary Fig.4b-d are provided as a Source Data file.

Supplementary Figure 5

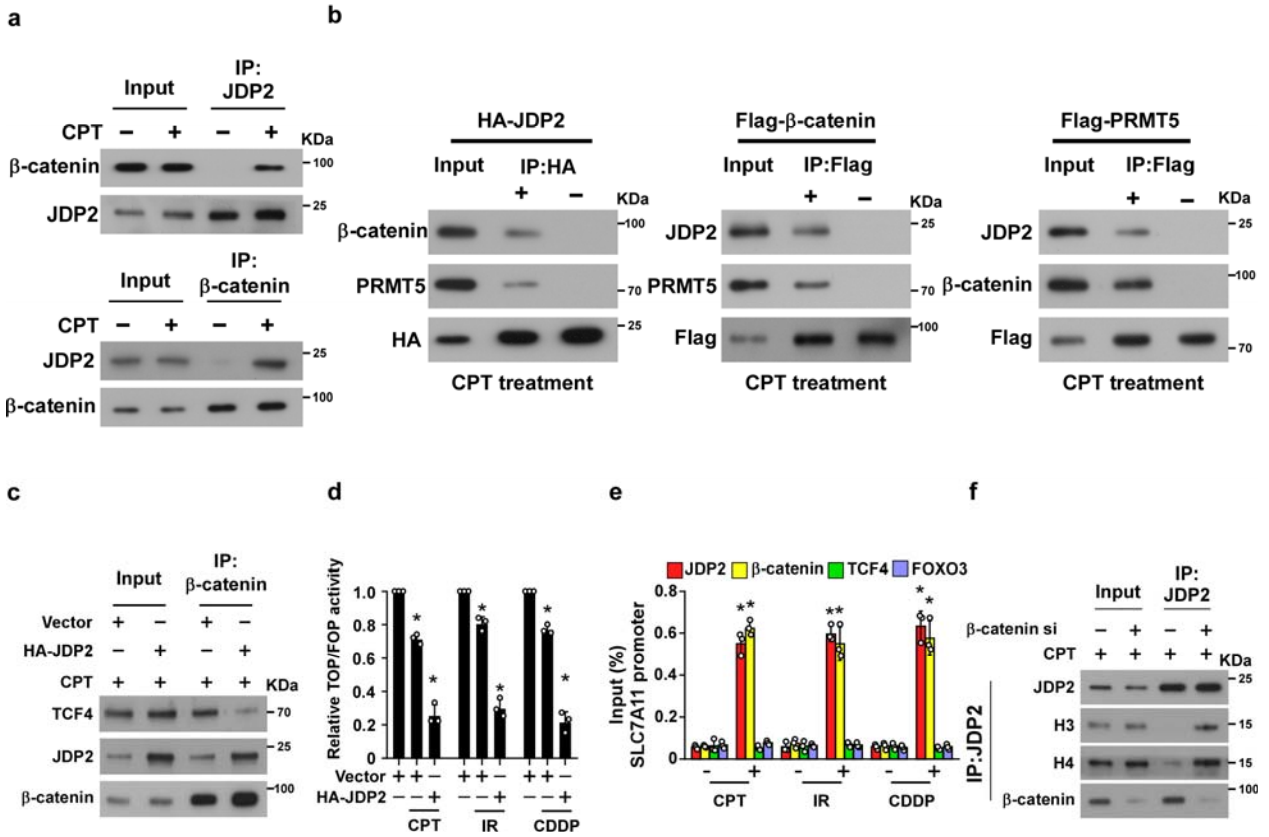

Supplementary Figure 5. β-catenin interacts with JDP2 in response to genotoxic stress. **(a)** IP assays using anti-JDP2 antibody (upper) and anti-β-catenin antibody (lower) were performed in cells treated with or without CPT (10 μM, 1 h), and IB analysis of expression of JDP2 and β-catenin. **(b)** IP assays using anti-HA or anti-Flag antibodies were performed in the indicated cells treated with or without CPT (10 μM, 1 h), and IB analysis of the expression of HA-tagged JDP2, or Flag-tagged β-catenin, or Flag-tagged PRMT5. **(c)** IP assays using anti-β-catenin antibody were performed in CPT (10 μM, 1 h)-treated cells transfected with HA-tagged JDP2, and IB analysis of the expression of TCF4, JDP2, and β-catenin. **(d)** Relative TOPflash or FOPflash luciferase reporter activity was analyzed in the indicated cells transfected with or without HA-tagged JDP2. **(e)** ChIP assays analyses of the enrichment of JDP2, or β-catenin, or TCF4, or FOXO3 on the promoter of *SLC7A11* in CPT (10 μM, 1 h)-, IR (10 Gy)-, or CDDP (10 μM, 1 h)-treated cells. **(f)** IP assays using anti-JDP2 antibody were performed in

CPT (10 μM, 1 h)-treated cells transfected with scramble or β-catenin siRNA, and IB analysis of the expression of JDP2, Histone 3, Histone 4, and β-catenin. Each error bar in d and e represents the mean ± SD of three independent experiments. * $P < 0.05$. Student' s 2-tailed t test. Source data of Supplementary Fig.5d-e are provided as a Source Data file.

Supplementary Figure 6

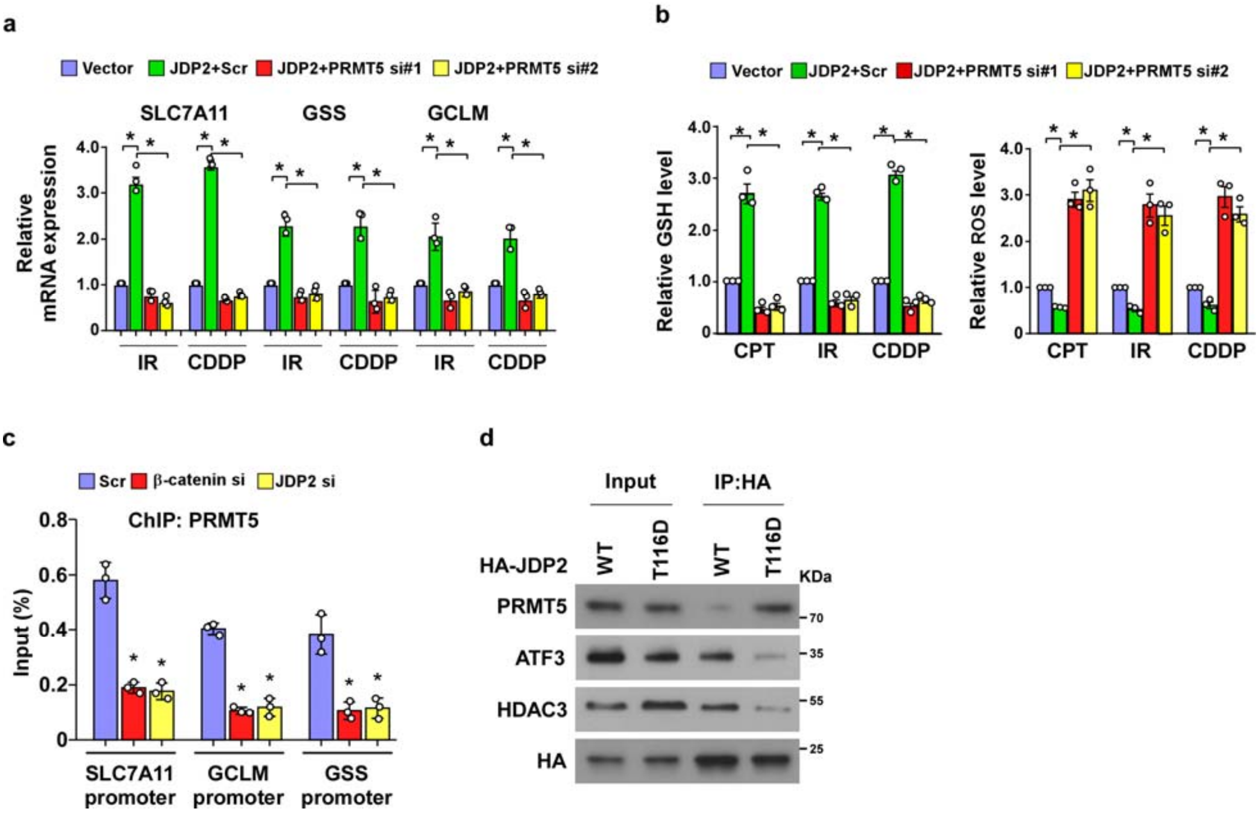

Supplementary Figure 6. PRMT5 promotes β-catenin/JDP2-activated GSH metabolism.

(a) Relative mRNA expression levels of *SLC7A11*, *GSS*, and *GCLM* genes in the indicated cells treated with IR (10 Gy) and CDDP (10 µM, 1 h), as quantified by qRT-PCR analysis. *GAPDH* served as the loading control. **(b)** Relative expression levels of GSH (left) and ROS (left) in the indicated cells treated with CPT (10 µM, 4h), or IR (10 Gy), CDDP (10 µM, 4h). **(c)** ChIP assays analyses of the enrichment of PRMT5 on the promoters of *SLC7A11*, *GSS*, and GCLM in the indicated cells treated with CPT (10 µM, 1 h). **(d)** IP assays using anti-HA antibody were performed in CPT (10 µM, 1 h)-treated cells transfected with HA-JDP2/wt or HA-JDP2/T116D mutant, and IB analysis of the expression of HA-JDP2, HDAC3, ATF3, and PRMT5. Each error bar in a-c represents the mean ± SD of three independent experiments. * $P < 0.05$. Student' s 2-tailed t test. Source data of Supplementary Fig.6a-c are provided as a Source Data file.

Supplementary Figure 7

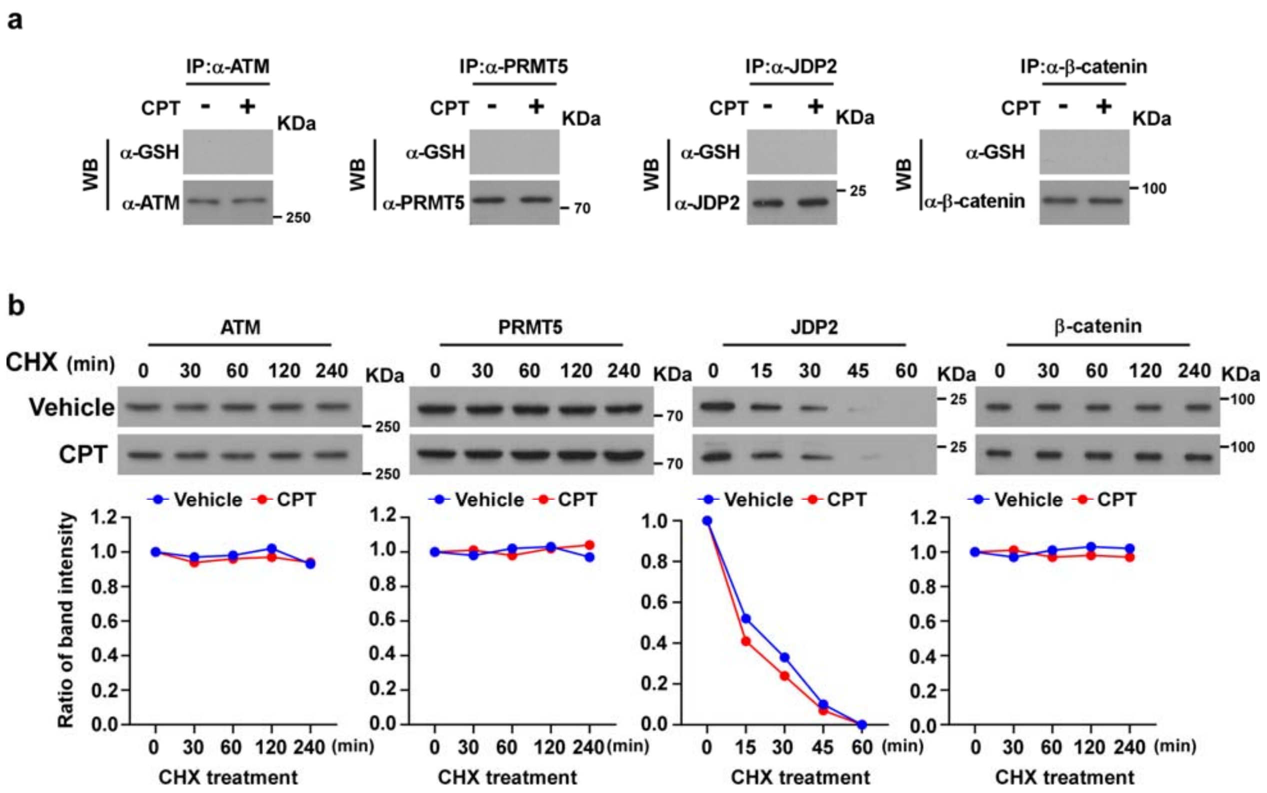

Supplementary Figure 7. Genotoxic stress did not induce either S-glutathionylational modification or stabilization of JDP2, β-catenin, ATM and PRMT5. (a) IP/IB analysis of S-glutathionylational modification of ATM, PRMT5, JDP2 and β-catenin in CPT (10 μM, 1 h)-treated cells. After immunoprecipitation (IP) with anti-ATM antibody, or anti-PRMT5 antibody, or anti-JDP2 antibody, or anti-β-catenin antibody, IB was further performed with anti-GSH antibody. **(b)** Upper: IB analysis of half-life of ATM, PRMT5, JDP2 and β-catenin protein in cells treated with cycloheximide (CHX, 40 μg/ml) plus vehicle or CHX (40 μg/ml) plus CPT (10 μM) at the indicated time. Lower: Ratio of band intensity of indicated protein in the cells treated with CHX plus vehicle or CHX plus CPT at the indicated time. Band intensity was quantified by densitometry and was represented as intensity relative to the zero-time point that set as 1.0. Source data of Supplementary Fig. 7b are provided as a Source Data file.

Supplementary Figure 8 Unprocessed scans of immunoblots shown in the figures.

Figure 1

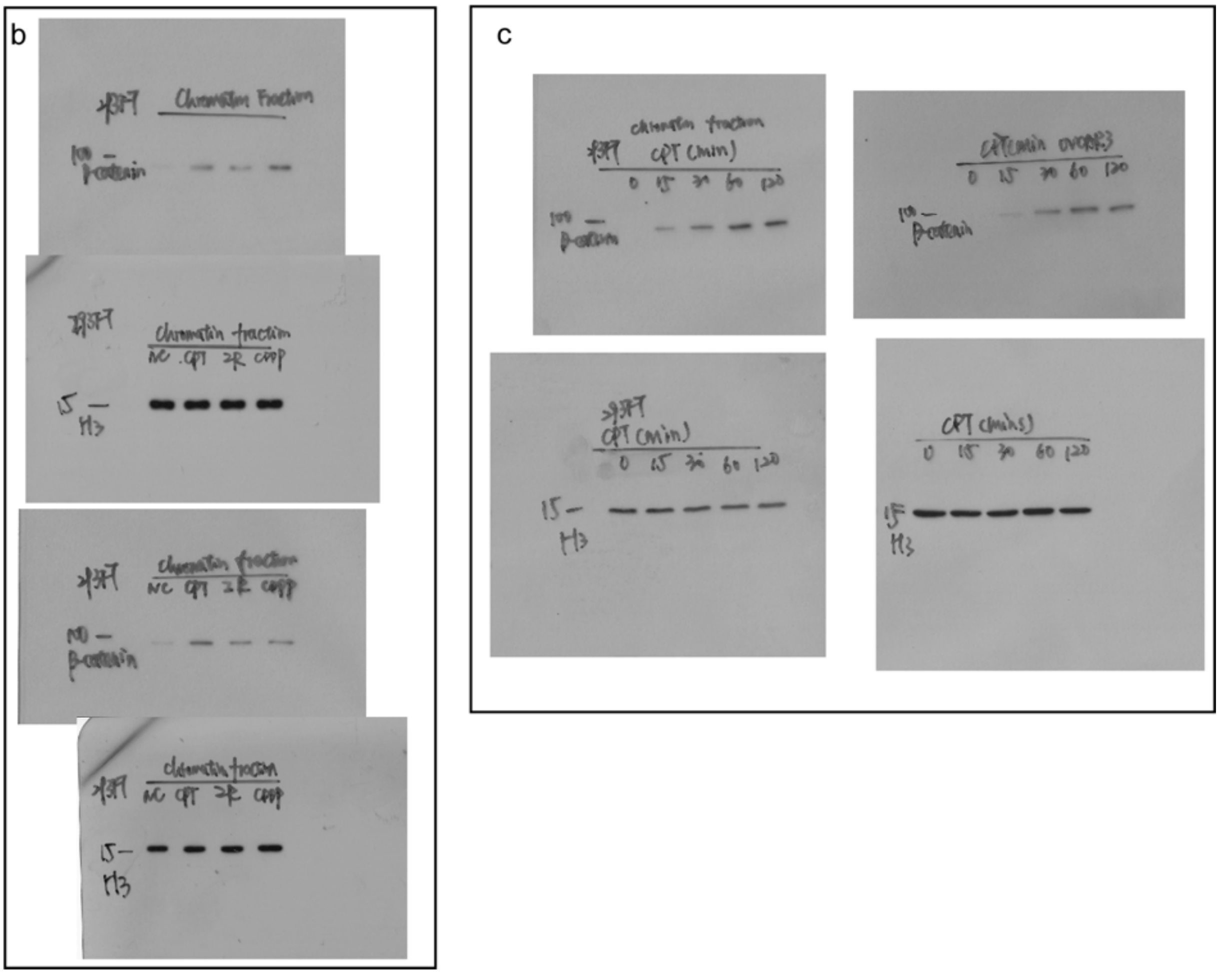

Figure 3

d

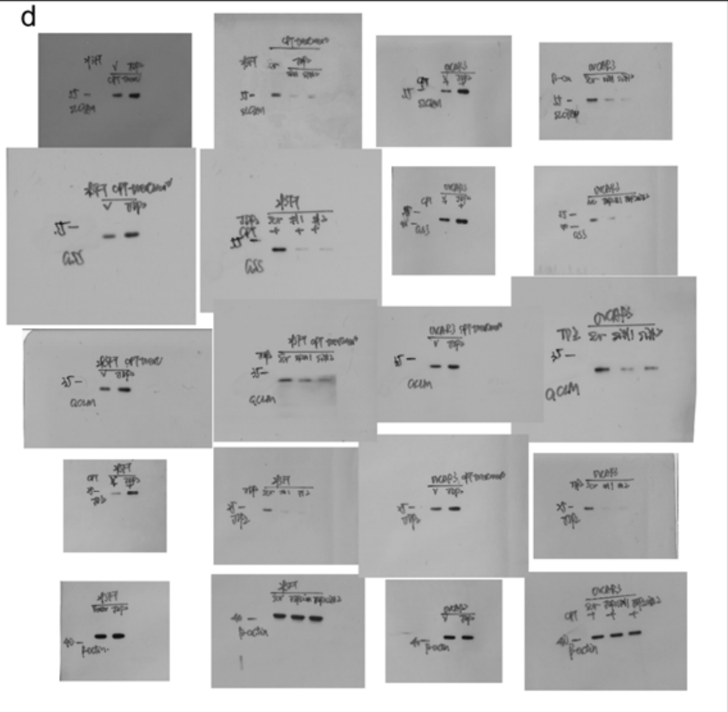

f

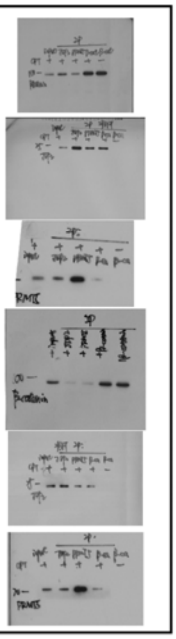

g

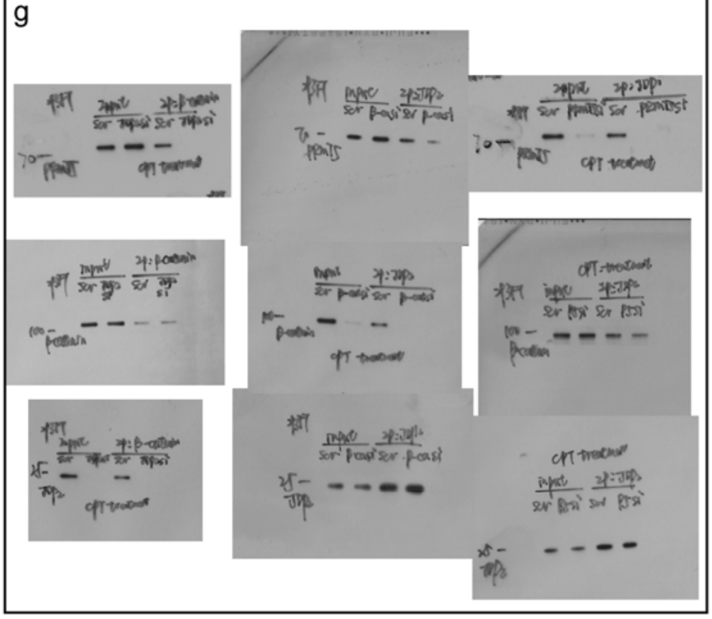

h

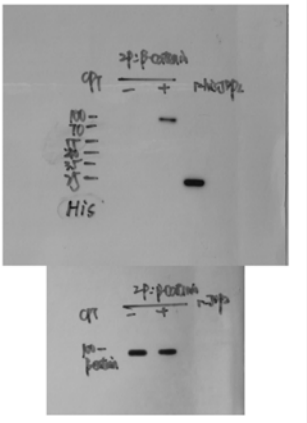

j

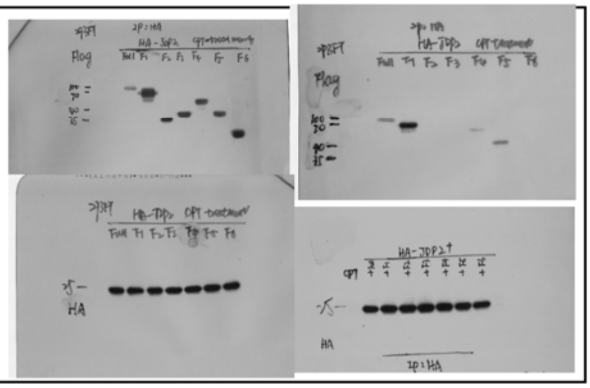

Figure 4

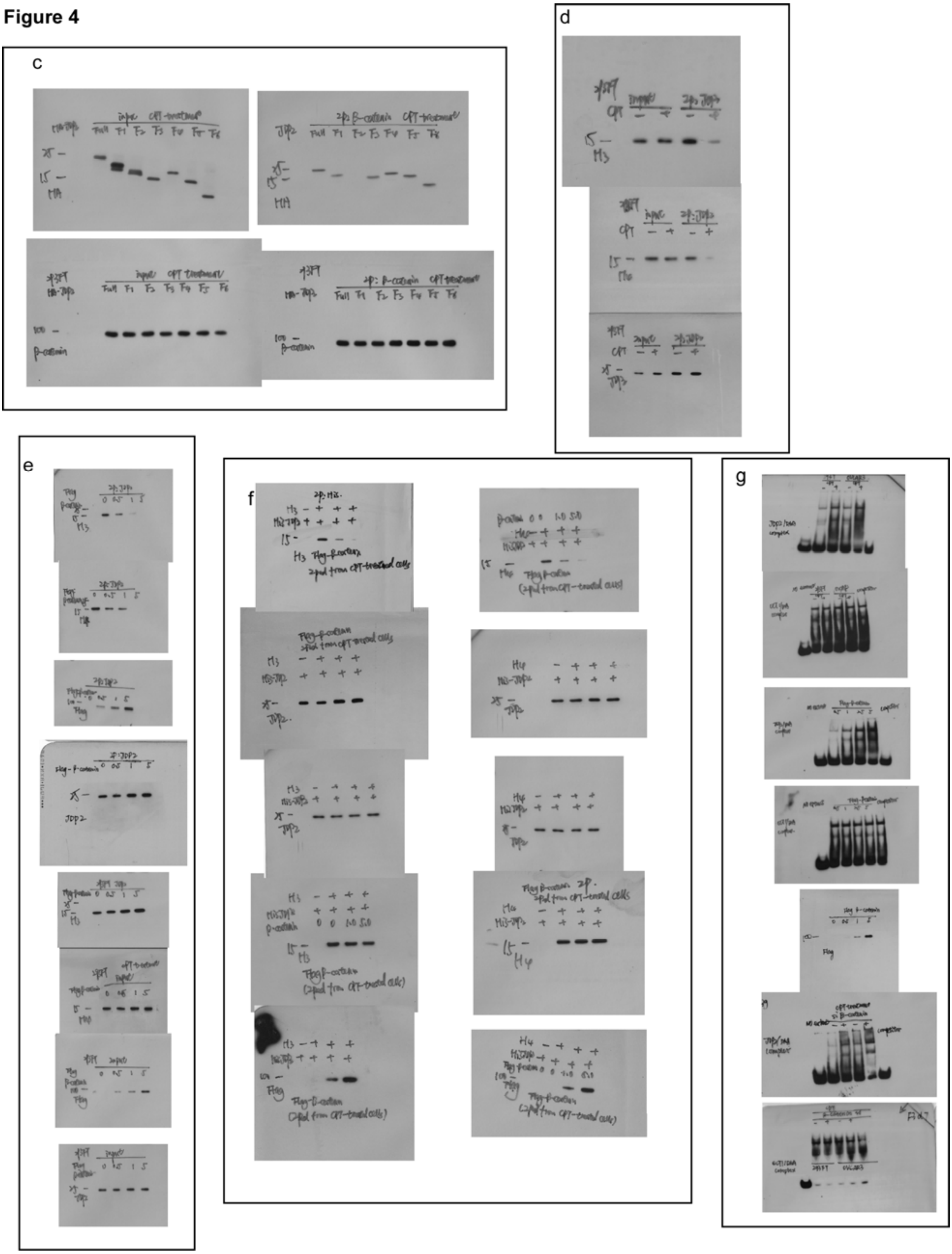

Figure 5

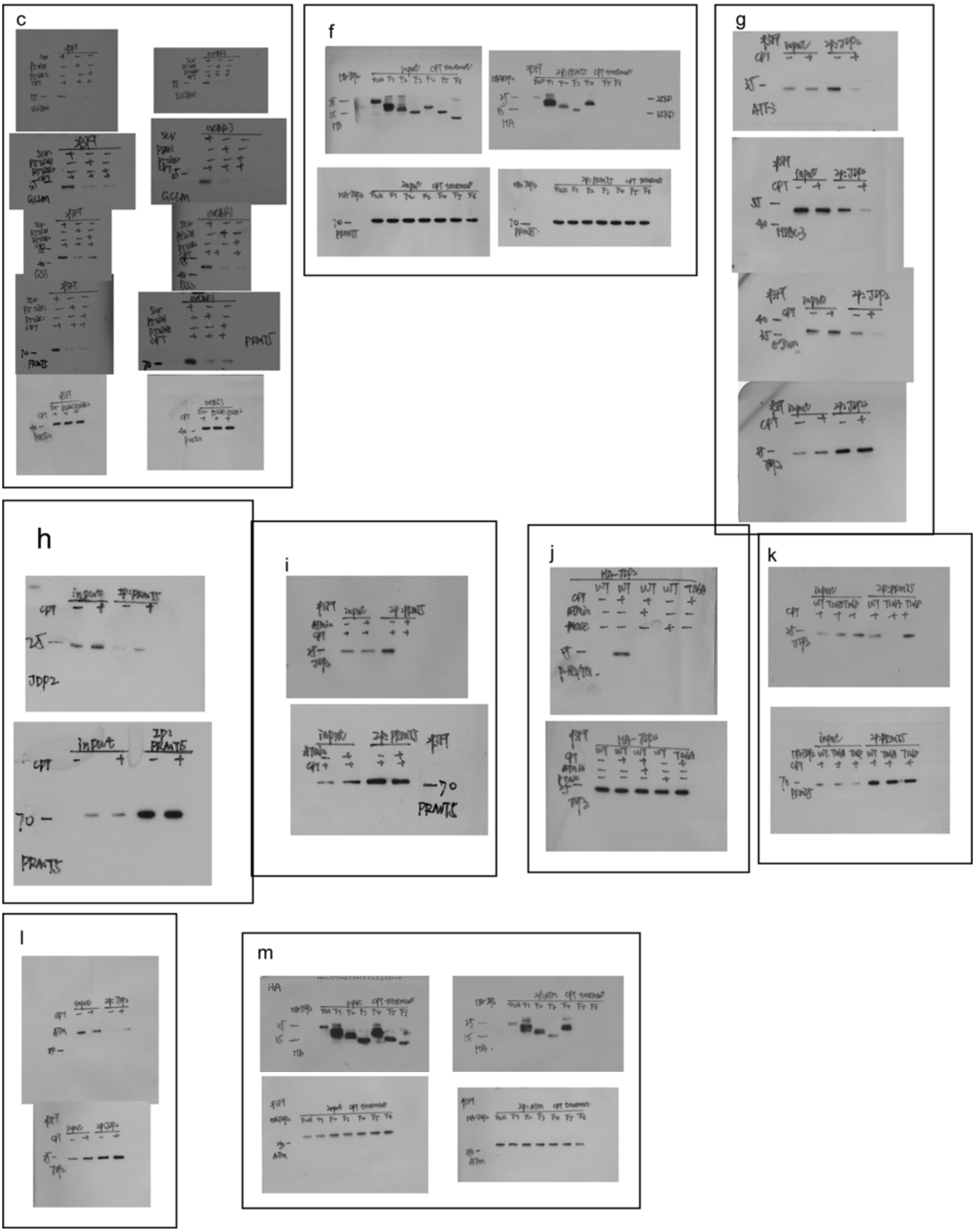

Figure 8

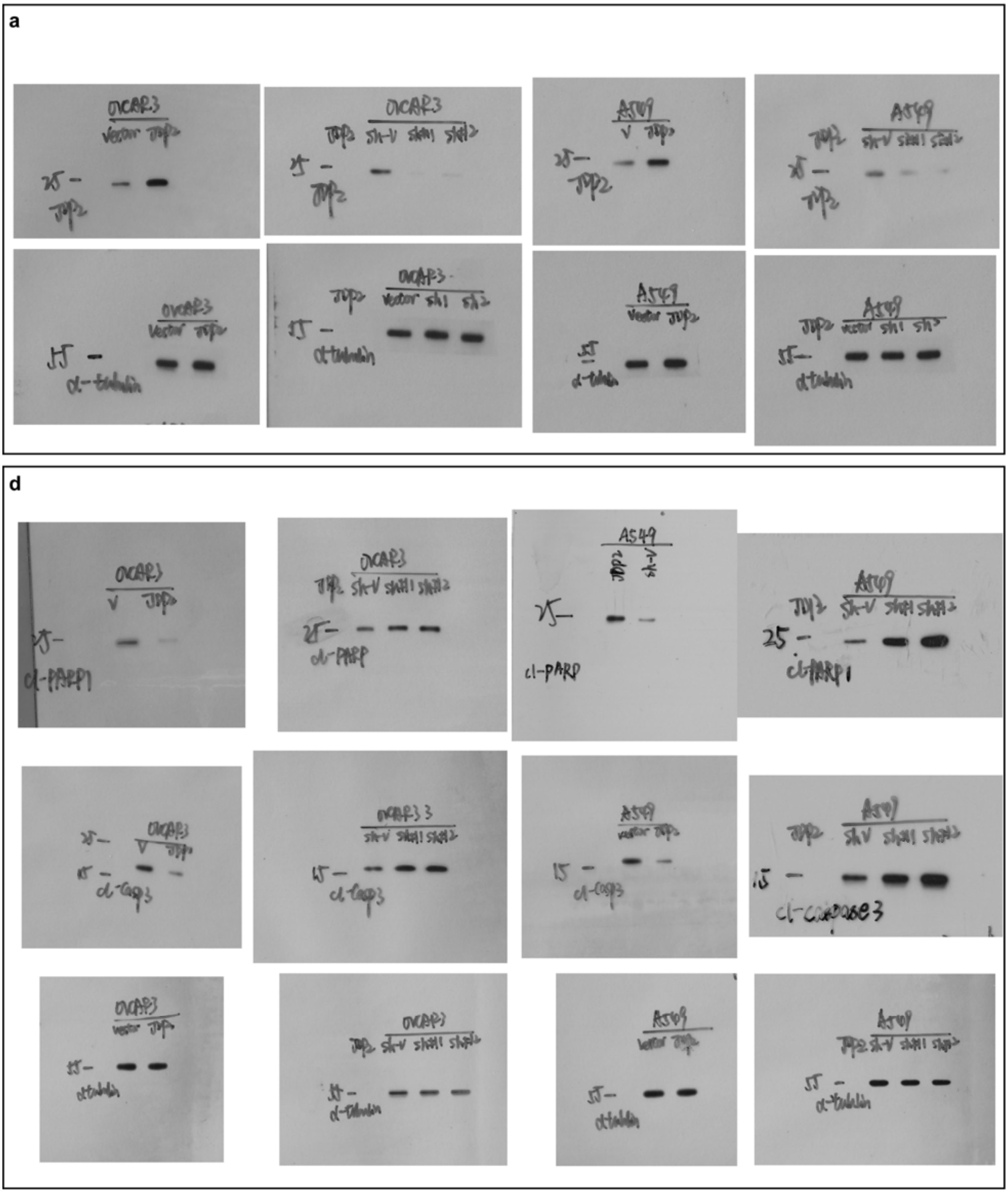

Figure 10

d

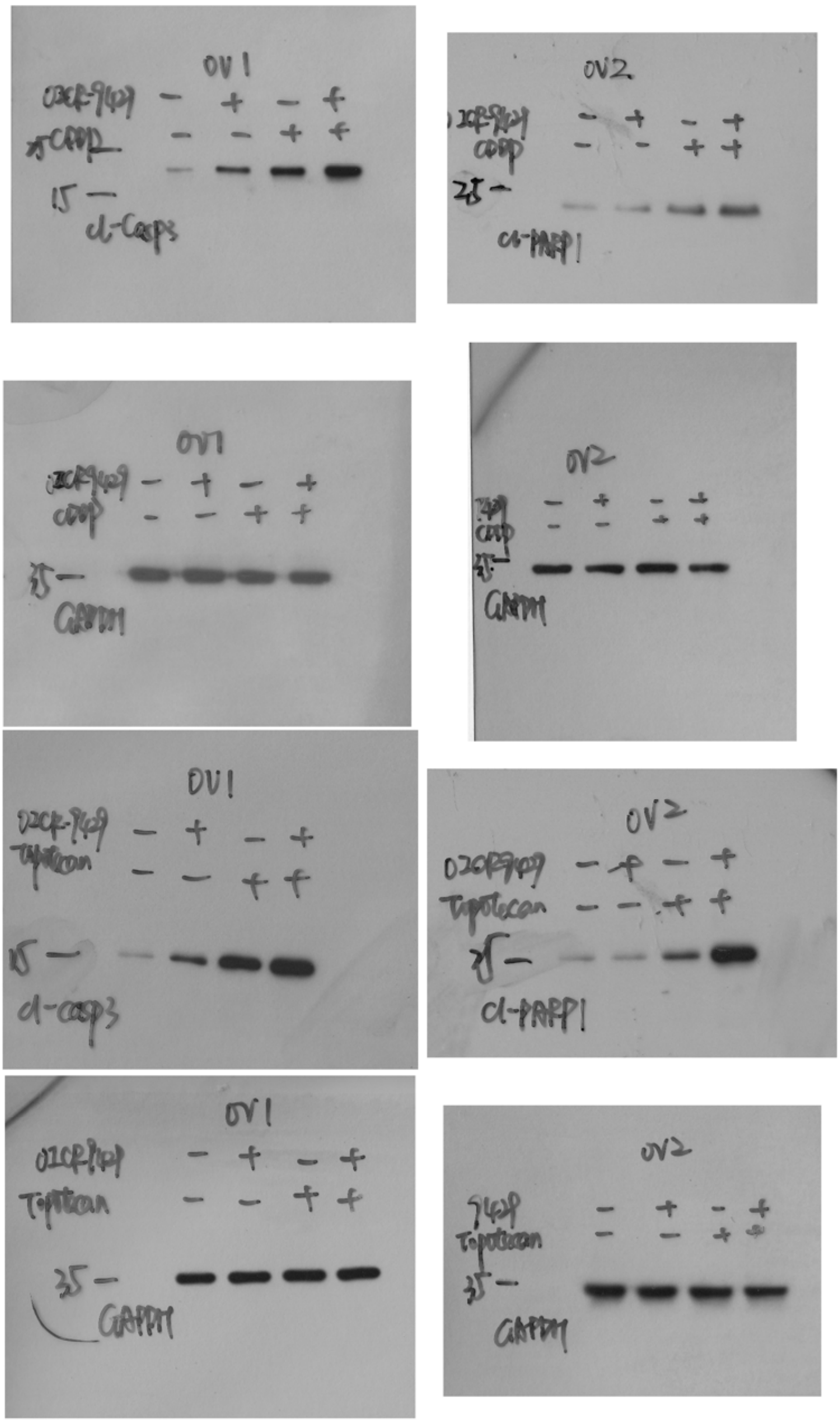

Supplementary Figure 1

b

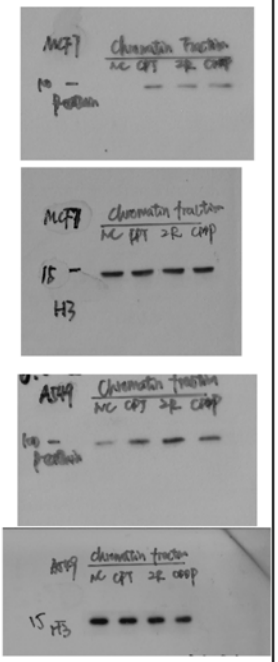

d

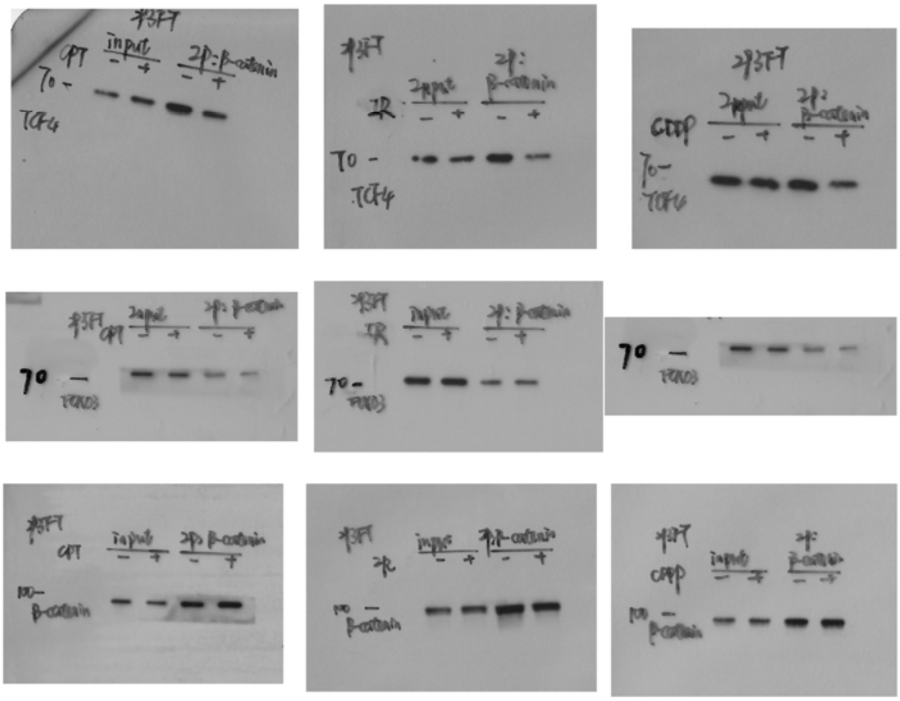

Supplementary Figure 2

e

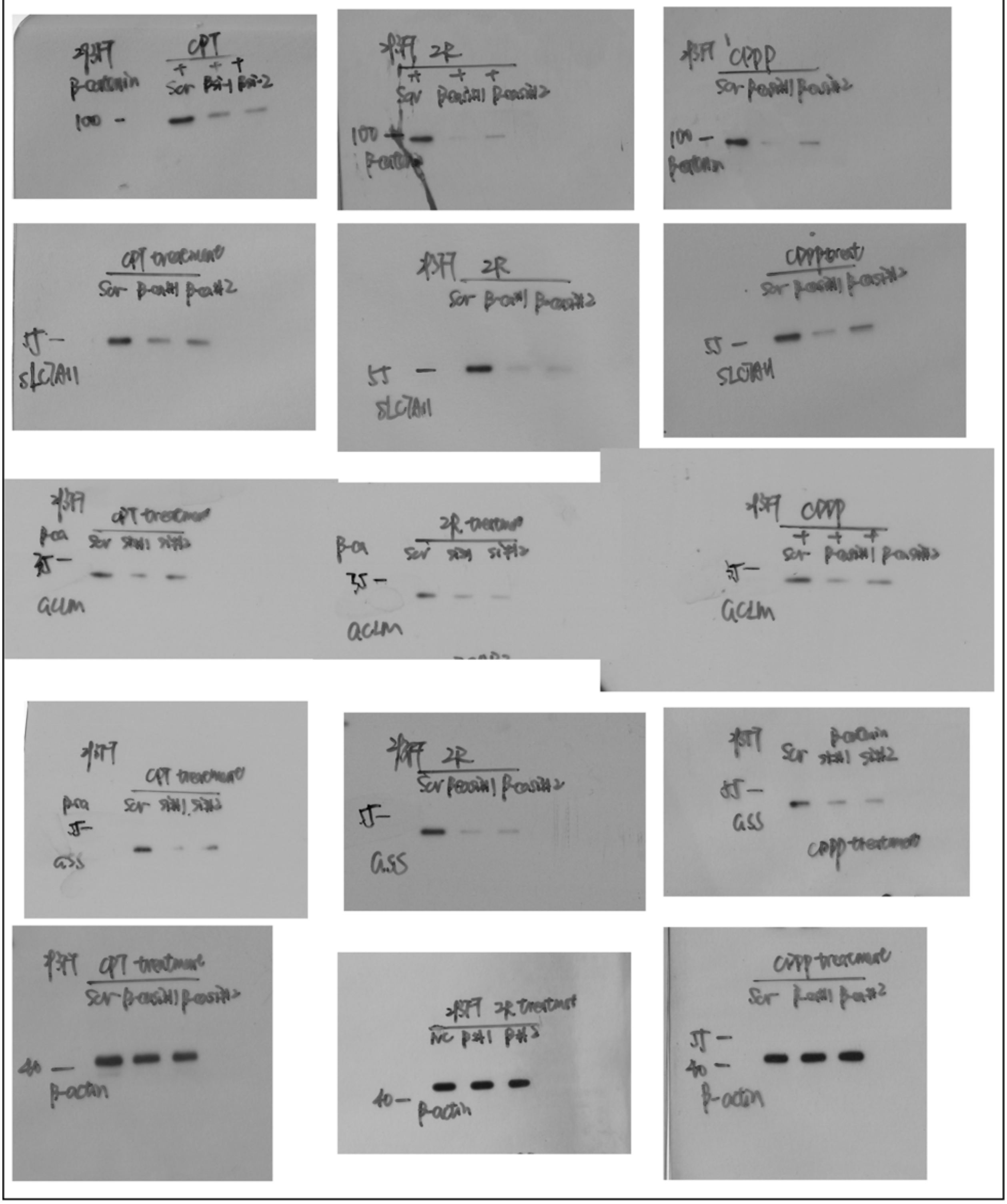

Supplementary Figure 3

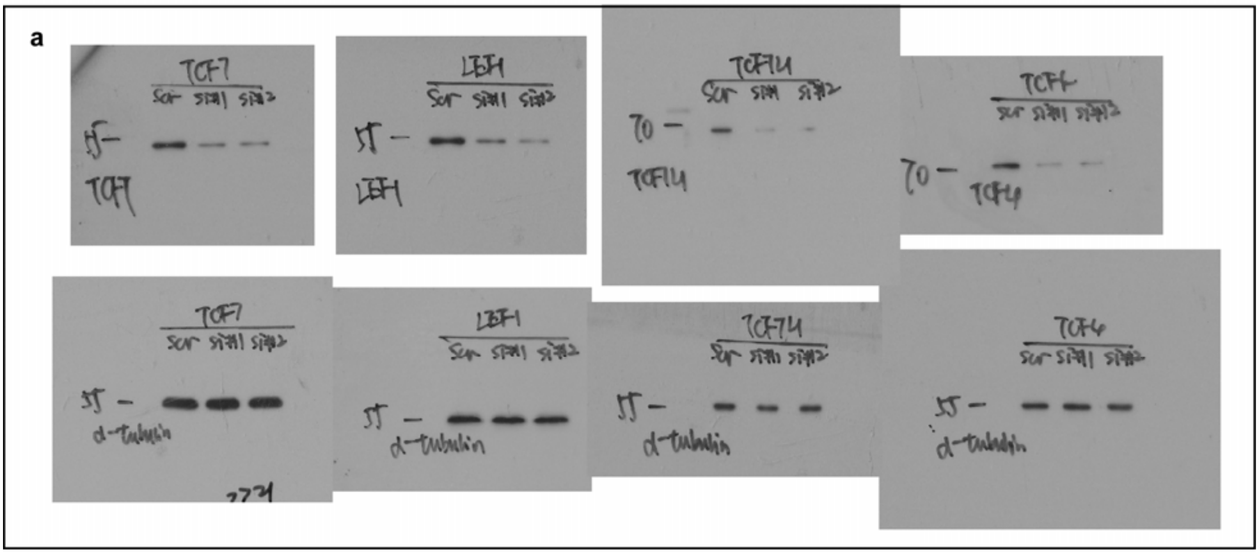

Supplementary Figure 4

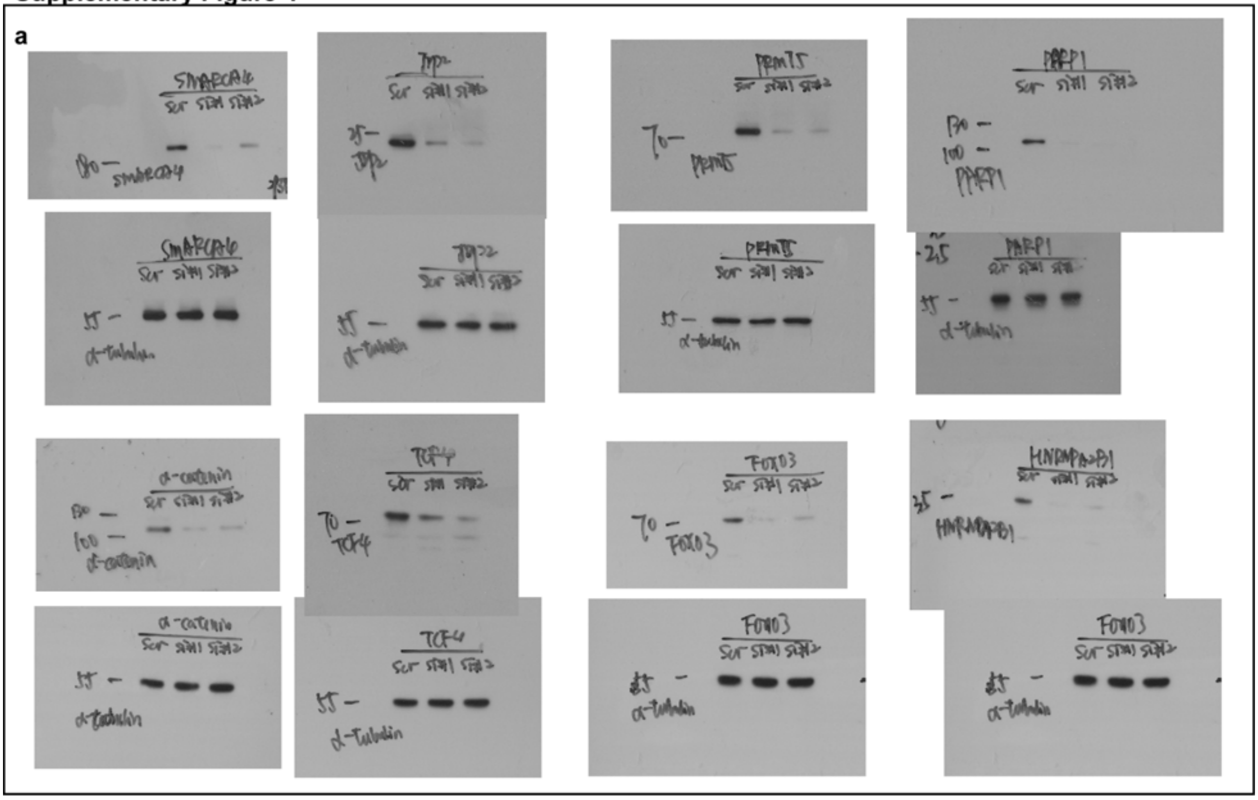

Supplementary Figure 5

a

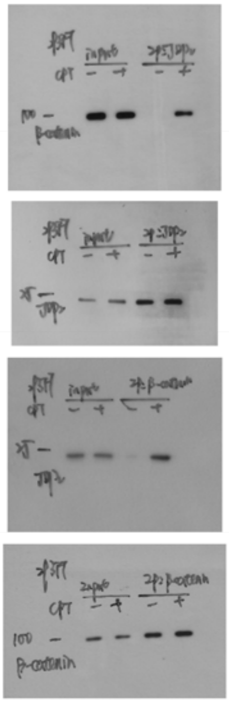

b

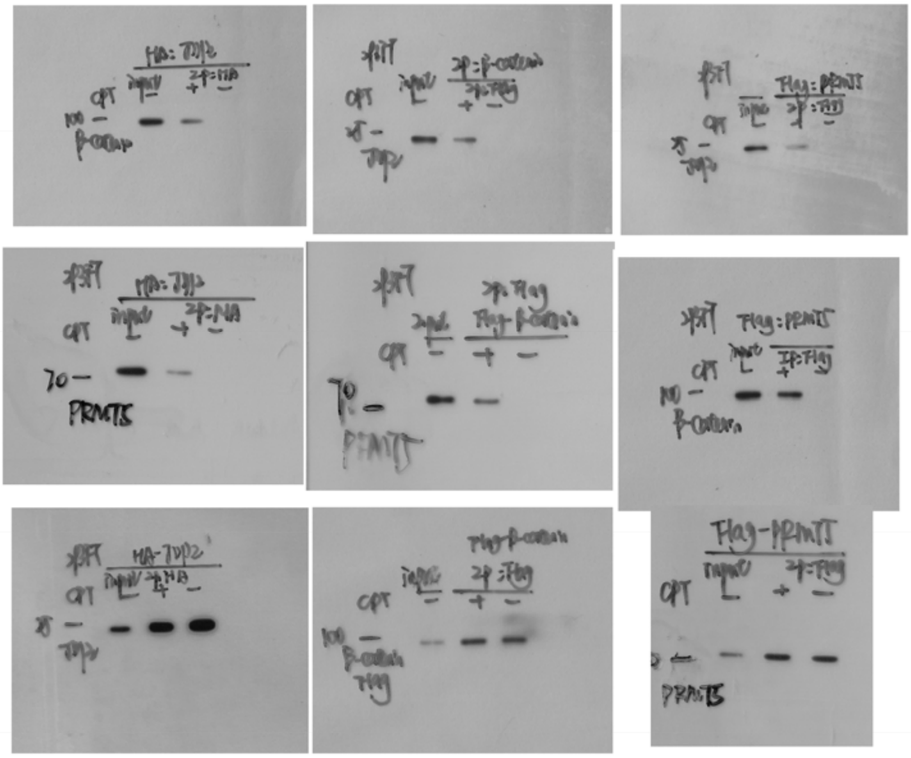

c

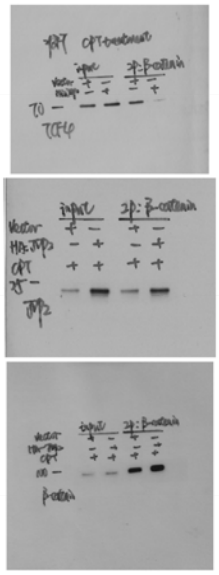

f

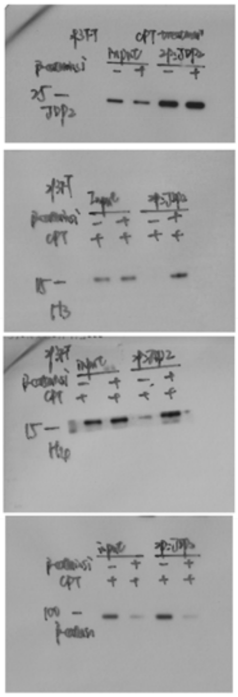

Supplementary Figure 6

e

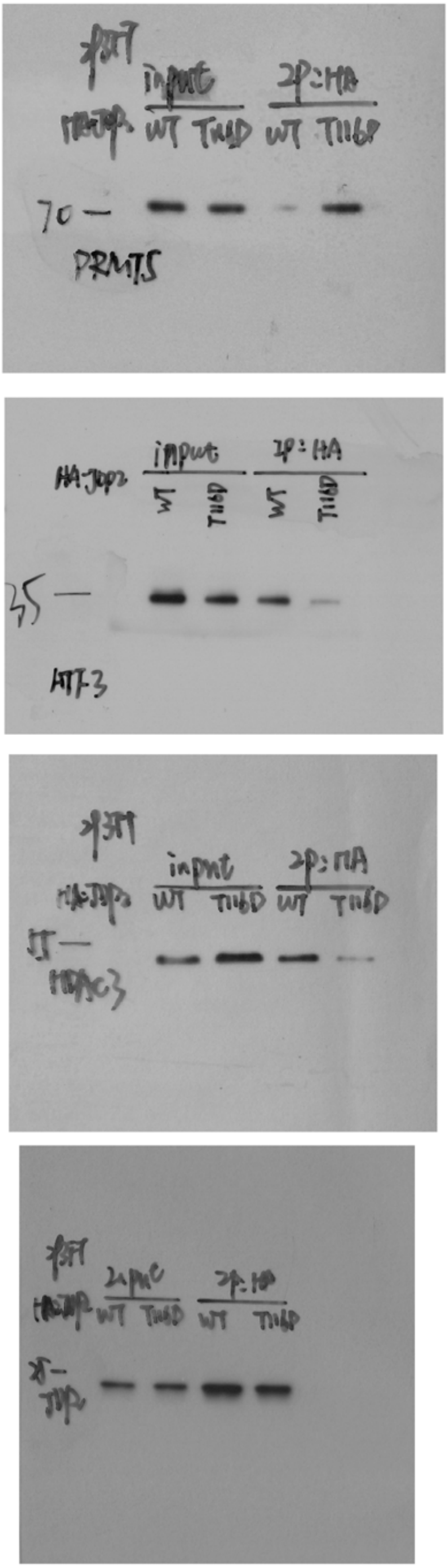

Supplementary Figure 7

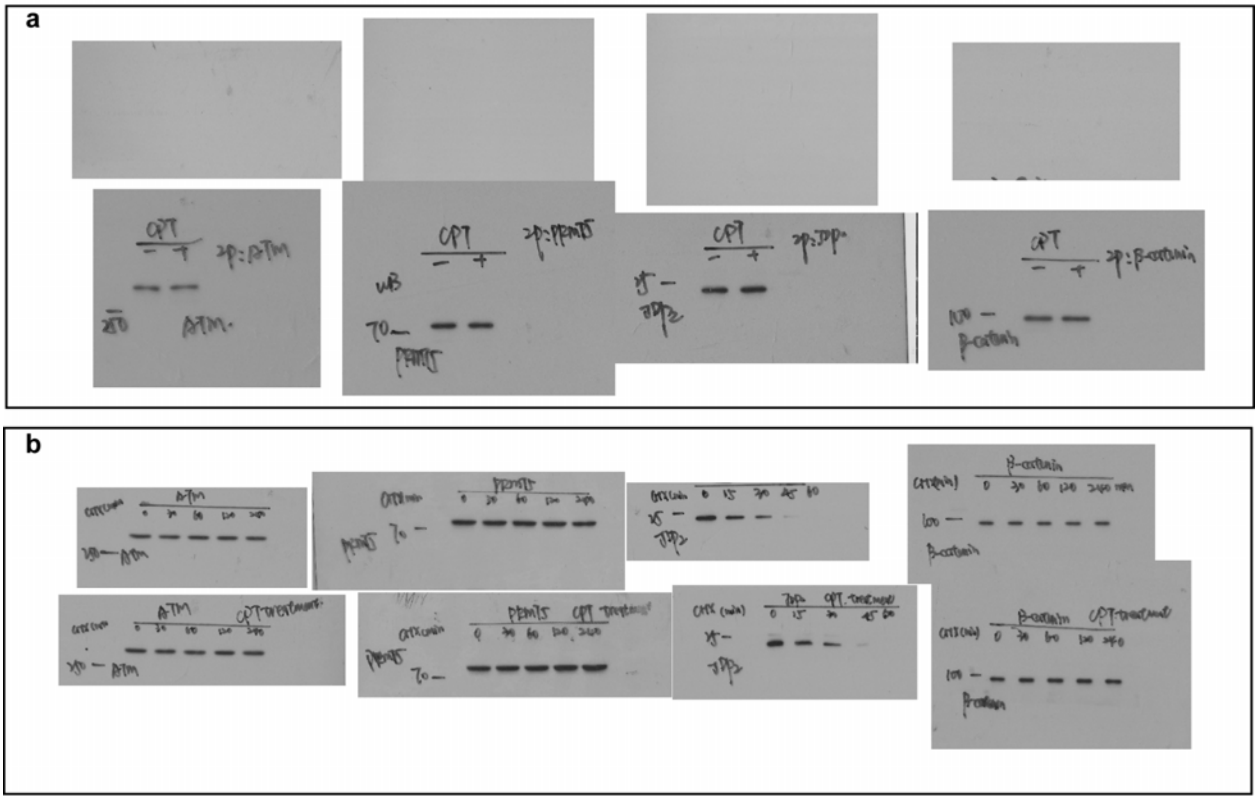

Supplementary tables

Supplementary Table 1. Analysis information of ChIP-seq

	ChIP (Rep1)	Input (Rep1)	ChIP (Rep2)	Input (Rep2)	ChIP (Pooled)	Input (Pooled)
Total Reads (M)	32.5	25.6	24.8	26.6	57.4	52.1
Mapped Reads (M)	31.7	24.7	23.9	25.5	55.6	50.2
Total Peaks	32709		19727		20521	
Promoter Peaks	8843		4936		6925	
5UTR Peaks	1163		394		688	
Exon Peaks	2125		653		1112	
Intron Peaks	11946		7895		6821	
3UTR Peaks	275		153		125	
TTS Peaks	733		358		365	
Intergenic Peaks	7273		5152		4265	
Pseudo Peaks	54		32		38	
Others Peaks	297		154		182	
Correlation analysis	$P < 1.0 \times 10^{-10}$, r = 0.85					

Supplementary Table 2. Clinicopathological characteristics and JDP2 expression in 146 patients with ovarian cancer.

Clinical features	Number	JDP2 expression		*P* values
		Low(n=64)	High (n=82)	
**Age (years)**				
≤ 62	71	33	38	0.531
> 62	75	31	44	
**Histological type**				
Serous	110	45	65	
Endometrioid	20	12	8	0.467
Mucinous	5	2	3	
Undifferentiated	11	5	6	
**FIGO stage**				
I/II	35	22	13	0.009
III/IV	111	42	69	
**Histologic grade**				
1	11	5	6	
2	28	13	15	0.94
3	107	46	61	
**Chemoresponse status**				
Chemoresistance	62	14	48	< 0.001
Chemosensitivity	84	50	34	
**Recurrence**				
Yes	115	43	72	0.004
No	31	21	10	
**Vital status**				
Alive	52	31	21	0.004
Dead	94	33	61	

Supplementary Table 3. Univariate and multivariate analysis of different prognostic parameters in patients with ovarian cancer by Cox-regression analysis.

	Univariate analysis		Multivariate analysis	
	P	**Hazard ratio (95% CI)**	***P***	**Hazard ratio (95% CI)**
**Age (years)**	0.116	1.388 (0.922-2.089)		
**Histological type**	< 0.001	1.553 (1.244-1.938)	0.002	1.422 (1.138-1.778)
**Histologic grade**	< 0.001	1.935 (1.349-2.774)	0.005	1.674 (1.166-2.402)
**FIGO stage**	0.002	2.249 (1.343-3.765)	0.060	1.672 (0.979-2.853)
**JDP2 expression**	< 0.001	2.319 (1.509-3.564)	0.001	2.105 (1.364-3.247)
