# Supplementary material for: Genotoxic stress-triggered β-catenin/JDP2/PRMT5 complex facilitates reestablishing glutathione homeostasis
Source: Nat Commun. 2019 Aug 21;10:3761. doi: 10.1038/s41467-019-11696-7 (PMC6704105; doi:10.1038/s41467-019-11696-7)
Supplement: Supplementary file 6 — Description of Supplementary data [file 41467_2019_11696_MOESM6_ESM.doc]

**Description of the Supplementary Data files for**

**Genotoxic stress-triggered β-catenin/JDP2/PRMT5 complex facilitates reestablishing glutathione homeostasis**

**Cao et al.**

**Supplementary Data 1.**  A list of potent β-catenin-binding proteins analyzed by immunoprecipitation (IP)/mass spectrometry (MS) assays.

**Supplementary Data 2.**  A list of corresponding peptides of each β-catenin-binding protein as presented in Supplementary Data 1.

**Supplementary Data 3.**  A list of all Primers and siRNA oligonucleotides.
